# Using a common data platform to facilitate audit and feedback on the quality of hospital care provided to sick newborns in Kenya

**DOI:** 10.1136/bmjgh-2018-001027

**Published:** 2018-09-19

**Authors:** Michuki Maina, Jalemba Aluvaala, Paul Mwaniki, Olga Tosas-Auguet, Catherine Mutinda, Beth Maina, Constance Schultsz, Mike English

**Affiliations:** 1 Health Services Unit, KEMRI-Wellcome Trust Research Programme, Nairobi, Kenya; 2 Department of Paediatrics and Child Health, University of Nairobi, Nairobi Kenya; 3 Nuffield Department of Medicine, University of Oxford, Oxford, UK; 4 Neonatal Unit, Pumwani Maternity Hospital, Nairobi, Kenya; 5 Department of Medical Microbiology, Academic Medical Centre, Amsterdam, Netherlands

**Keywords:** neonatal care, audit and feedback, quality of care

## Abstract

Essential interventions to reduce neonatal deaths that can be effectively delivered in hospitals have been identified. Improving information systems may support routine monitoring of the delivery of these interventions and outcomes at scale. We used cycles of audit and feedback (A&F) coupled with the use of a standardised newborn admission record (NAR) form to explore the potential for creating a common inpatient neonatal data platform and illustrate its potential for monitoring prescribing accuracy. Revised NARs were introduced in a high volume, neonatal unit in Kenya together with 13 A&F meetings over a period of 3  years from January 2014 to November 2016. Data were abstracted from medical records for 15 months before introduction of the revised NAR and A&F and during the 3 years of A&F. We calculated, for each patient, the percentage of documented items from among the total recommended for documentation and trends calculated over time. Gentamicin prescribing accuracy was also tracked over time. Records were examined for 827 and 7336 patients in the pre-A&F and post-A&F periods, respectively. Documentation scores improved overall. Documentation of gestational age improved from <15% in 2014 to >75% in 2016. For five recommended items, including temperature, documentation remained <50%. 16.7% (n=1367; 95%  CI 15.9 to 17.6) of the admitted babies had a diagnosis of neonatal sepsis needing antibiotic treatment. In this group, dosing accuracy of gentamicin improved over time for those under 2  kg from 60% (95%36.1 to 80.1) in 2013 to 83% (95% CI 69.2 to 92.3) in 2016. We report that it is possible to improve routine data collection in neonatal units using a standardised neonatal record linked to relatively basic electronic data collection tools and cycles of A&F. This can be useful in identifying potential gaps in care and tracking outcomes with an aim of improving the quality of care.

**Summary box**

- Improving information systems that support routine monitoring of quality and outcomes at scale is an important part of efforts to enhance neonatal care.
- We highlight clinical data elements that are poorly recorded by practitioners in routine settings, findings that can help revise the standardised record form.
- It is possible to improve routine data collection and prescribing accuracy in neonatal units using a standardised neonatal record linked to relatively basic electronic data collection tools and cycles of audit and feedback to improve medical care.

## Introduction

Newborn deaths account for approximately 44% of under-five deaths globally,^1^ largely attributable to preterm birth, sepsis and intrapartum complications.^2^ Specific interventions such as newborn resuscitation, thermal care, use of oxygen and early recognition and treatment of neonatal infections have been identified as major interventions to reduce neonatal deaths that can be effectively delivered as part of basic hospital services.^3^ However, there are few data on whether such interventions are delivered in routine settings in low-income and middle-income countries (LMIC). Available evidence suggests that adherence to recommended forms of care is poor.^4^ For example, looking at treatment of neonatal infections, in a recent assessment of neonatal units in Kenyan hospitals, more than 20% of the prescriptions of gentamicin were above safe, recommended doses.^5^

An ability to monitor routine antibiotic prescribing is also aligned with increasing concern over antimicrobial resistance (AMR). Neonatal units including intensive care units can be hotspots for development and transmission of antimicrobial-resistant organisms.^6^ This may be due to the extensive empirical use of antimicrobial agents, inappropriate choice of antibiotics, inappropriate dosing and extended duration of administration coupled with poor infection prevention and control practices.^7–9^ Spread of antibiotic-resistant organisms among neonates may subsequently be manifest in increased length of hospital stay, increased hospital costs and greater morbidity and mortality.

Unfortunately, poor documentation, record keeping and information systems^10^ preclude effective monitoring of both the delivery of effective interventions in general and the use of antibiotics in particular. This is worsened by limited human resource capacity, equipment and supplies.^11^ Improving information systems that support routine monitoring of quality and outcomes at scale is therefore an important part of efforts to enhance neonatal care in LMIC.^12^

To address the challenge of inadequate record keeping and routine monitoring, we worked with a high volume, low-resource neonatal unit in Kenya using audit and feedback (A&F). This was coupled with efforts to develop/update a newborn admission record (NAR) that could support a common inpatient neonatal data platform for monitoring care and outcomes at scale. A&F is based on the premise that if clinicians are informed of what is not consistent with required practice, they will change behaviour. However, effects of A&F as an improvement strategy have been varied with some reporting very modest or no effects.^13^ A&F may be more effective if based on data that are valid and timely^14^ with recent and prior work suggesting effectiveness is enhanced if coupled with other interventions such as the use of standardised admission records.^15^

Here, we report on the effects of repeated cycles of A&F linked to use of standardised record forms on the completeness of patient level information in a LMIC neonatal unit. We illustrate the potential value of better data for monitoring quality and prescribing accuracy and we identify which clinical data elements are poorly recorded by practitioners in routine settings, findings that can help revise the standardised record form.

### Context: Pumwani Maternity Hospital

Pumwani Maternity Hospital, the largest public maternity hospital in Kenya, is located in the capital city Nairobi. The hospital, which serves mainly the urban poor population, conducts approximately 22 000 deliveries annually; the newborn unit has approximately 4500 admissions annually and holds close to 60 babies each day. Care is overseen by four consultant paediatricians supervising a team of six medical officers and four clinical officers (non-physician clinicians) typically working so that there are 2-3 clinicians in every shift. Nursing care is provided by registered nursing officers, a minority of whom have specialised neonatal training. Typically, only 2–3 nurses are on duty per shift assisted by trainee nurses. The research team had no role in patient care but did support the provision of one clerk to collect data daily.

## Description of routine data collection using a NAR

A NAR promoting documentation of key patient characteristics at the time of admission was originally developed in 2006 as part of the Emergency Treatment and Triage plus admission approach which includes skill training in essential inpatient newborn care.^16 17^ Adoption of this NAR has been at the discretion of hospital teams with modest uptake but a suggestion that it can improve data availability.^5^ In Pumwani, there was an effort to revise the NAR in 2014 with the local team so it might better capture important information, for example, maternal human immunodeficiency virus (HIV) status, length of gestation and mode of delivery. The NAR is divided into different sections which include: (1) relevant maternal history, (2) babies’ biodata and clinical history, (3) babies’ examination findings and admission vital signs, (4) the basic laboratory tests ordered and (5) primary and secondary diagnosis on admission. The clinical variables included are based on the key signs and symptoms that national guidelines recommend should be assessed for all sick newborns.^18 19^ A minimum data set (used for the national reporting system) is collected on all patients, while a full data set including all clinical and treatment data is collected on an automatically generated random sample of 60% of admitted newborns. Table 1 shows the data collected from the NAR that were used for the analysis in this report and some of the variables that were added after the modification of the NAR in 2014. The complete NAR is attached in the online supplementary appendix 1.

10.1136/bmjgh-2018-001027.supp1 Supplementary data

**Table 1:** NAR variables used for analysis

Domain	Number of variables	Variables included in the analysis
Maternal history*	6	Mothers age, parity, gravidity, mothers blood group, HIV status, VDRL status.
Demographics and diagnosis	12	*Admission information*: date of birth, admission date, gender, birth weight, age in days, gestational age, mode of delivery, APGAR score at 5 min, admission diagnosis. *Discharge information*: date of discharge or death, outcome (dead or alive), diagnosis on discharge or death.
Presenting complaints	6	Presence of fever, convulsions, difficulty in breathing, vomiting, difficulty feeding, apnoea.
Cardinal signs on examination	4	Grunting, central cyanosis, bulging fontanelle, floppy (inability to suck, reduced movements/activity).
Other physical examination	18	*Airway*: stridor.* *Breathing*: bilateral air entry on chest examination*, crackles on chest examination, chest indrawing. *Circulation*: skin pinch, femoral pulses*, capillary refill time, heart murmur*, pallor, peripheral to central skin temperature gradient.* *Others*: signs of eye infection, signs of umbilical infection, presence of skin rashes/pustules, stiff neck, irritability, jaundice, gestational size*, severe wasting.*
Vital signs	4	Temperature, respiratory rate, heart rate, oxygen saturation.*

*Variables included in the modified NAR after 2014.

NAR, newborn admission record; HIV, human immunodeficiency virus; VDRL, venereal disease research laboratory

Electronic data capture from the medical records which are in paper form (NAR, treatment sheets) occurs at discharge. Every working day data were collected and managed using Research Electronic Data Capture (REDCap) electronic data capture tools. REDCap is a secure, web-based application designed to support data capture for research studies.^20^ These data are abstracted by a trained clerk stationed at the health facility. To ensure data quality, built-in range and validity checks are employed at the point of data entry; an automated error checking procedure is run daily on site with corrections made. Further error checks are performed by the data management team after de-identified data are synchronised to a central server. A more comprehensive description of the data cleaning and data quality assurance process is described elsewhere.^21^

### Implementation activities

For a period of 15 months from January 2013 to March 2014, the period before the A&F process, we collected baseline data in a retrospective survey. Data were collected from records that included an earlier form of the NAR already in use at the hospital. The records examined were from a random selection of dates across the 15 months with the intention of capturing data on approximately 55 admissions per month. From April 2014, the research team worked with the neonatal unit clinical team to revise the NAR tailoring it to the needs of the hospital, a process resulting in the addition of further variables (maternal history, addition of clinical signs and symptoms, addition of admission diagnosis, table 1). The hospital team introduced the revised NAR into its preprinted medical files to make it a routine medical record filled for all admissions. A&F was used to highlight documentation of key variables showing how the NAR was used and the completeness of documentation. A&F was integrated into existing monthly mortality meetings organised by the hospital teams and attended by the clinical, nursing teams and hospital management where these data were presented quarterly. Areas for improvement were found and actions to promote change identified—with responsibility for leading these left to the paediatricians and the clinical team.

## Experience before and after A&F

The period for these data was divided into two phases, the baseline data collection period January 2013 to 31 March 2014 and the period with feedback from April 2014 to November 2016. We defined the 6-months periods, two pre-A&F and six post-A&F, to explore any effect of efforts to improve documentation. For each patient, each variable within an analysis domain was assigned a binary score denoting availability of documentation (ie, 0=No; 1=Yes). We then calculated, for each analysis domain, the percentage of documented variables among the total number of variables that could be documented for the patient population over the 6-month period. The percentage of completed documentation was plotted with 95% CI to examine changes and trends over time.

To understand what documentation tasks remain difficult despite A&F, we calculated, for each individual variable, the percentage of patients with missing documentation for that variable over the last 6-month interval (ie, April–November 2016) and stratified items as poorly (25%–50%) or very poorly (<25%) documented.

We provide documentation trends for individual variables that are indispensable to delivering appropriate drug dosing to newborns–namely, birth weight and gestational age–and illustrate the impact of improved documentation on trends of gentamicin posology over time to illustrate the potential monitoring value of better data. The Kenyan guidelines recommend a once daily gentamicin dose of 3 mg/kg for those babies <2 kg and 5 mg/kg for those ≥2 kg in the first 7 days of life.^19^ We considered a correct dose to be within a ±20% margin of error. Summary statistics and data visualisation were conducted in R statistical software V.1.0.136.

NARs were examined for 827 patients in the pre-A&F period (January 2013–March 2014). The revised NAR was examined for 7336 patients in the A&F period, over six consecutive 6-month intervals (n=1067 (Period 1, 2014), n=1941 (Period 2, 2014), n=1144 (Period 1, 2015), n=1103 (Period 2, 2015), n=1230 (Period 1, 2016) and n=680 (Period 2, 2016)). The last 6-month period in 2016 had lower patient numbers due to a 14-day doctors strike in the month of October. Of the 7985 patients included, 46% (n=3656) were female, 78.3% (n=6251; 95% CI 77.6 to 79.4) were admitted on the date of birth, 90.5% (n=7207; 95% CI 89.8 to 91.1) were admitted within 48 hours of delivery and 9% (n=712; 95% CI 8.3 to 9.6) of the admitted babies died. The mean birth weight on admission was 2.88 kg (95% CI 2.86 to 2.90).

There were 13 A&F feedback meetings between January 2014 and November 2016, eight specific to A&F feedback and five as part of monthly morbidity and mortality meetings.

### Documentation across domains

**Figure 1:**
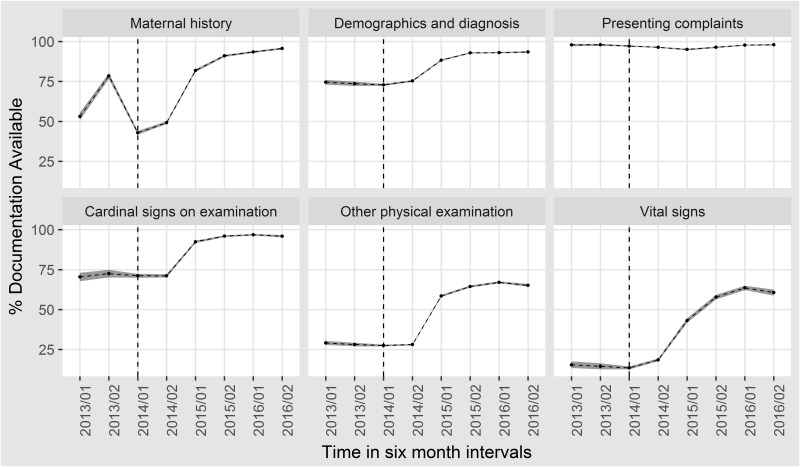
Trends in completeness of documentation of various parts of the newborn admission record over 3 years. The black shade around the trends is the 95% CIs around the estimates. The dotted line represents the introduction of prospective data collection in April 2014.

The documentation across all domains showed improvement over time and after introduction of the revised NAR as shown in figure 1. The greatest improvement was noted in the domains that had low baseline performance at the onset. Thus, documentation of babies’ vital signs, maternal history and other physical examination showed greatest improvement. Although documentation of vital signs improved by more than 50% between 2014 and 2016, performance stagnated at less than 75%, as it did in another domain ‘other physical signs’ (table 1).

In the variable-specific analysis over the last 6-month period (April–November 2016), we explored documentation of 57 variables in 680 patients. Variables with poor (25%–50%) documentation were general examination of the skin (other clinical signs) and admission temperature (vital signs). Those with very poor documentation (<25%) were all under other physical examination; these were documentation of visible wasting (a possible indicator of intrauterine growth retardation), skin pinch and skin temperature (signs of neonatal dehydration) and the presence of femoral pulses and eye discharge. This variable-specific analysis helps explain the plateauing seen in figure 1.

### Documentation of gestational age and birth weight

**Figure 2:**
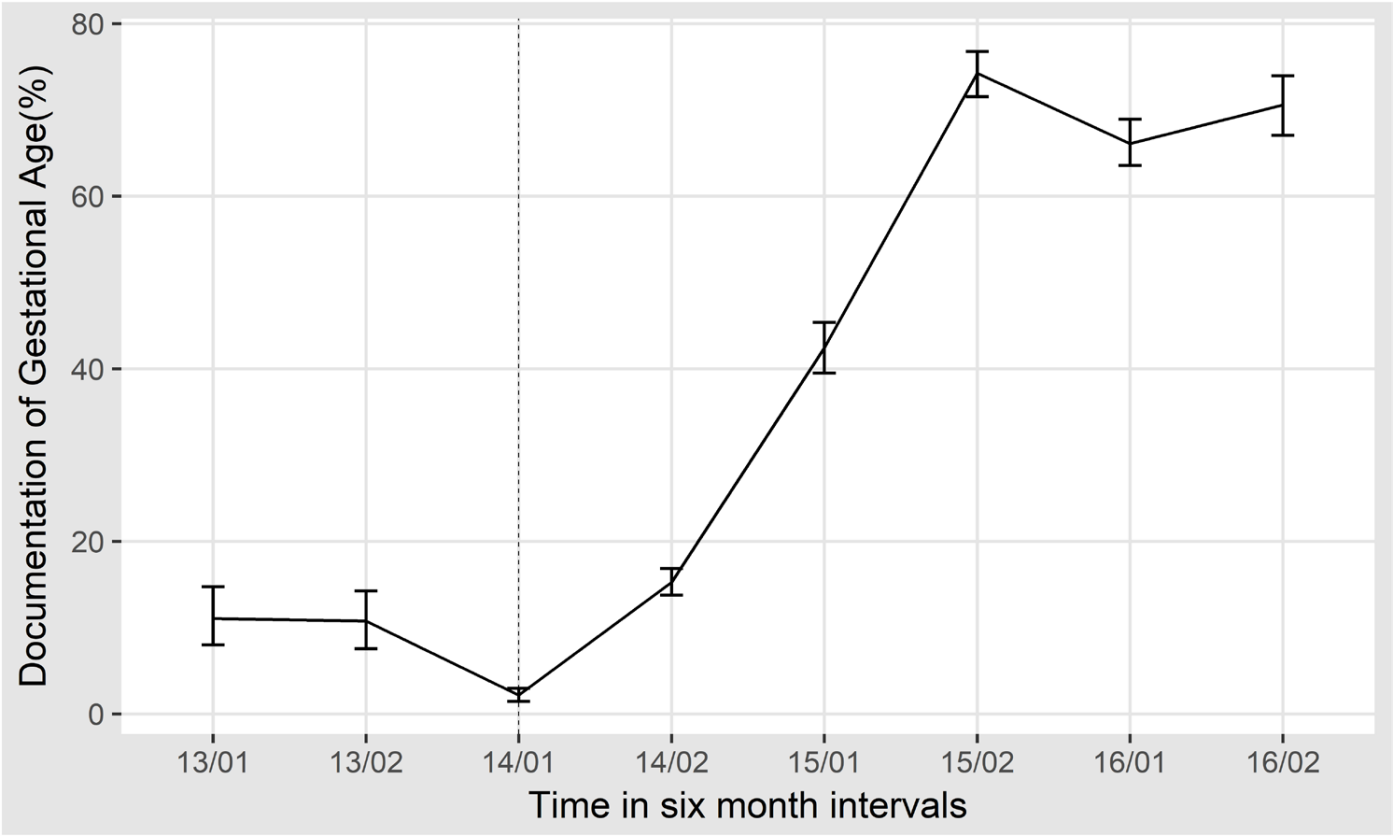
Proportion of documentation of gestational age among hospitalised neonates in 6-month intervals with 95% CIs around the estimates. The dotted line represents the introduction of prospective data collection.

Documentation of birth weight and gestational age are important in feed and antibiotic prescribing decisions and other aspects of newborn care. The documentation of birth weight remained consistently high with >95% documentation since 2013. Documentation of gestational age has shown gradual improvement over the data collection period from <15% documentation in 2014 to >75% documentation in 2016 as shown in the figure 2 below.

### Dosing of gentamicin

There were 16.7% (n=1367; 95% CI 15.9 to 17.6) of the admitted babies with a diagnosis of neonatal sepsis needing antibiotic treatment during the study period. Of these neonates diagnosed with neonatal sepsis, 81.4% (n=1113; 95% CI 79.2 to 83) were prescribed a combination of penicillin and gentamicin (national first line). An additional 39.5% (n=2688; 95% CI 38.4 to 40.7) of admitted neonates were put on antibiotic treatment without a clear diagnosis of neonatal sepsis who typically had respiratory distress, prematurity or asphyxia; these prescriptions were predominantly penicillin and gentamicin.

**Figure 3:**
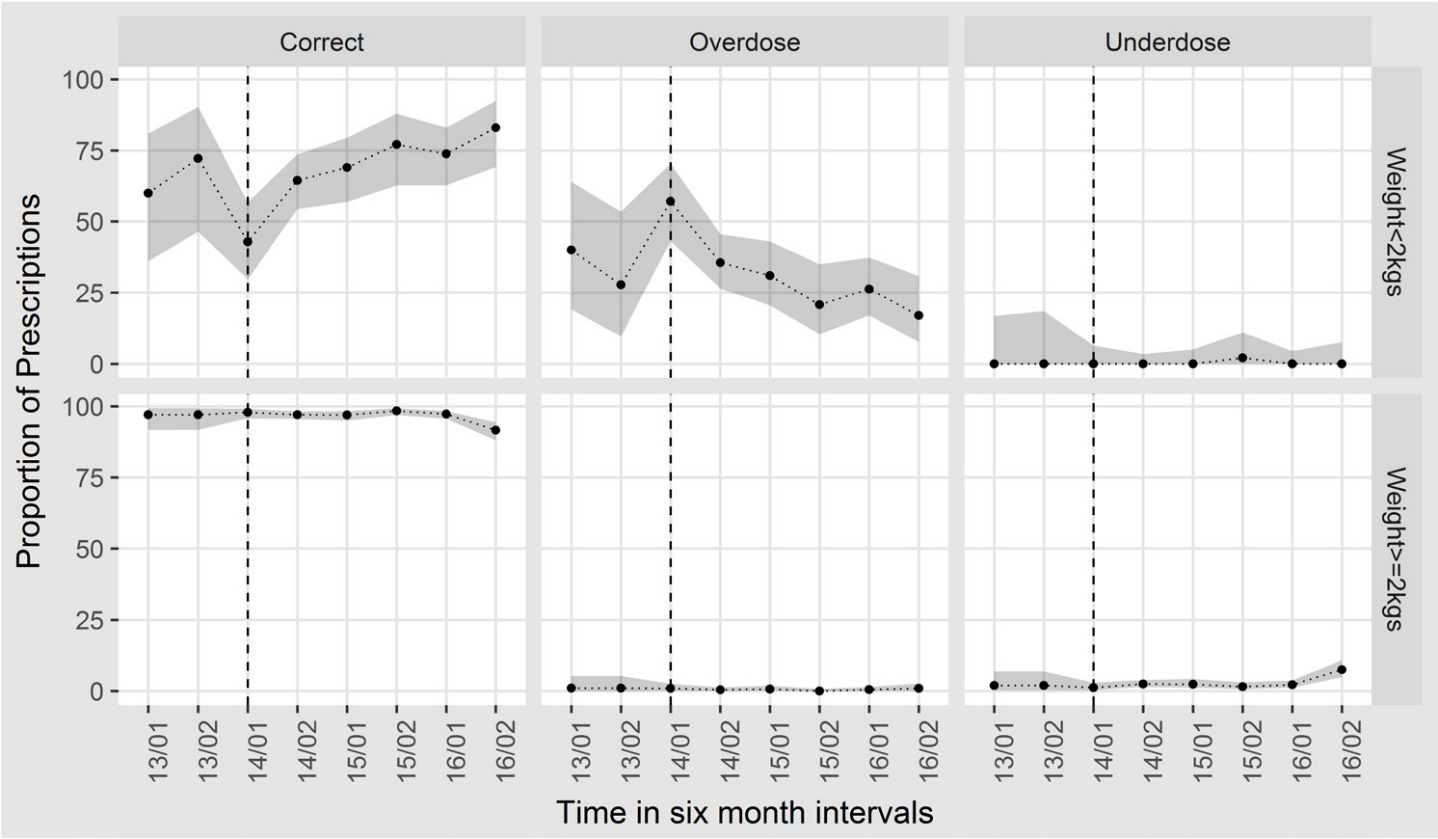
Charts showing dosing trends of gentamicin over time in 6-month intervals from January 2013 for those under 2 kg (Upper panel) or over 2 kg (lower panel). The grey shade indicates the 95% CIs around the estimates. The dotted line represents the introduction of prospective data collection.

Of the neonates who received gentamicin, 4.4% (n=161; 95% CI 3.8 to 5.1) received an overdose and 2.4% (n=86; 95% CI 1.9 to 2.9) were underdosed. By plotting trends by weight, the major changes were noted in those <2 kg with a reduction in the proportion of overdoses of gentamicin prescribed as shown in the figure 3 below.

## Impact of introducing the NAR with A&F cycles

We set out to improve availability of clinical data on neonatal admissions in a large Kenyan hospital. Here we report how repeated cycles of A&F were used to improve availability of information, share lessons on information that is difficult to collect in busy routine clinical settings and illustrate how such data may be of potential value for monitoring quality of care taking the example of gentamicin dosing. We focused on reinforcing the use of a standardised admission record as these have been associated with more thorough documentation in some LMIC settings but not previously, to our knowledge, in neonatal care.^22^ We report good documentation in six domains that include 50 specific variables. However, five specific variables were typically poorly documented by clinicians; examination of the skin (colour, bruising, pustules), admission temperature, visible wasting, skin pinch and skin temperature. Clinicians may feel some of these are less relevant to the babies’ clinical condition on the day of birth (wasting, skin pinch and skin temperature) when most admissions occur as these signs are typically associated with later onset neonatal illnesses. With thermal care being an essential aspect of neonatal care, we note that temperature is still recorded poorly with approximately 25%–50% documentation. Documentation of gestation was noted to improve across the period to >75% documentation. This is much better than previous reports from Kenyan hospitals.^5^ Here, we also report some improvement in the accuracy of gentamicin dosing for neonates under 2 kg. Dosing errors including antibiotics occur more among sick neonates compared to any other population; these errors may have a more significant effect as neonates have little physiological ability to buffer these errors.^23^ Most of these errors occur in the prescribing phase as compared with the dispensing and administration phase. Internationally, various interventions have been instituted to reduce these prescriptions errors.^24–26^ Our data demonstrate that it is possible to track correctness of dosing over prolonged periods in a busy hospital setting.

A&F as an improvement tool has been widely used in clinical settings but evidence on its effects is mixed.^13^ It may be more effective if the performance targeted has large room for improvement. This was sometimes the case in our setting where, for example, documentation of gestation was <20% in the baseline period of our study. It has also been noted that A&F is more effective when the targeted change is less complex (requiring no specific skills) and compatible with clinician norms and values.^27^ Having the organisational buy-in and involving the leadership in goal and target setting also makes the process of A&F more effective.^28^ The goal setting and feedback meetings in our setting were attended by the heads of the units and hospital managers which ensured that the process was in line with overall hospital priorities.

Paucity of newborn data in terms of type of care provided, morbidity and mortality is still a major challenge in many facilities.^29^ The WHO names actionable information systems as a key pillar in provision of quality maternal and neonatal care. This allows clinicians to make timely and appropriate decisions.^12^ There is increasing interest at the global level in tracking quality of care at scale, particularly care provided to the newborn.^30^ A central element of monitoring quality of care at scale is a common data set. Our work demonstrates that clinicians providing care in a busy, routine hospital setting can be encouraged to use standardised neonatal record forms with high levels of documentation especially for variables that are clinically meaningful to them. Establishing an agreed and standard medical record could enable neonatal networks to be formed with an aim of improving care provided to patients. In such networks, colleagues may also share experiences and can provide a ‘bottom up’ method of problem solving^31^ helping improve clinical outcomes at scale.^32^ With the realisation that quality data may improve care provision, many governments and hospitals in Africa are moving towards electronic medical records (EMR).^33^ This may provide an opportunity to integrate agreed common data elements as part of an EMR as well as improve the value of existing District Health Information systems.^34^

Limitations of this approach are that documentation only captures some aspects of quality and that information can be documented incorrectly or activities recorded but not done. However, data from case records remain the most feasible form of information to collect on patients at scale. The accuracy of data is also hard to verify for certain types of data. For example, most of the documentation of gestation is still based only on maternal history rather than ultrasound dating in Kenya. Other challenges to implementing better information systems remain; the resources needed to support data capture and analysis with better information are often given low priority in resource allocation. While data collection for this project was partly supported by the research team, this was limited to the A&F reports and the modest costs for data collection (clerk costs). However, there are discussions with the ministry of health and the national paediatric association on how best to transition and expand these activities to ensure continuity beyond the current funding. Despite this being a research project, outputs from this work feed into the routine mortality and morbidity meetings and monthly health information reports further reinforcing data use and institutionalisation of the activities beyond the current funding cycle.

## Conclusion

It is possible to improve routine data collection in neonatal units using a standardised neonatal record linked to relatively basic electronic data collection tools. These tools could collect data on significant presenting complaints, maternal history, babies’ physical examination, investigations and treatment. Data collected on such a platform at wider scale can be useful in identifying potential gaps in care with an aim of improving the quality of care provided in facilities and tracking outcomes. Monitoring antibiotic use could be especially valuable in the current era. Implementing such systems takes time and needs significant support from clinicians, nurses and hospital managers.
