# Supplementary material for: Using a common data platform to facilitate audit and feedback on the quality of hospital care provided to sick newborns in Kenya
Source: BMJ Glob Health. 2018 Sep 19;3(5):e001027. doi: 10.1136/bmjgh-2018-001027 (PMC6150140; doi:10.1136/bmjgh-2018-001027)
Supplement: Supplementary data [file bmjgh-2018-001027supp001.pdf]

Appendix

Neonatal Admission Record

Newborn Unit Admission Record

**Baby's details**										
**Baby Name**			**Date of Admission**	dd/mm/yyyy		**IP No.**				
**DOB**		**Age**	days	hrs	**Sex**	F □	M □	**Birth Wt**	kg	
**Gestation**	weeks	**Temp (°C)**		**Apgar**	1m	5m	10m	**Wt now**	kg	
**BBA?**	Y □	N □	***..if Yes born where***	□ Home	□ Clinic	□ Other hospital				
**Delivery**	SVD	Vacuum	Breech	CS	**Resuscitation**	None	Oxygen	Bag/Mask		
If CS reason	□ Elective	□ Emergency	**Placenta**							
**ROM**	<12h	12-18h	>18h	**Given Vitamin K**	Y□	N□	**Given Eye prophylaxis**	Y□	N□	

**Mother's details**									
**Name**		**Age**		**IP No.**					
**Residence sublocation**		**Gravidity**		**Parity**					
**HIV**	□ **Pos** □ **Neg**	**ARVs**	□ **Yes** □ **No**	**VDRL**	□ **Pos** □ **Neg**	**Blood Gp**			
**Fever**	Y□	N□	**Antibiotics**	Y□	N□	**Diabetes**	Y□	N□	**TB treat** Y□ N□
**Labour**	**1st Stg** hr	**2nd Stg** mins	**Hypertension**	Y□	N□	**APH**	Y□	N□	

Babies Presenting Problems / Mothers Problems & relevant drugs pre-admission

**Vital Signs**	**Temp (°C)**		**Resp Rate**	bpm	**Pulse**	/min	**O2 Sat**	%

Time baby seen	am/pm			Stridor	Y□	N□
Length of illness	days			Cry	□Normal □Weak	□Hoarse
Fever – No. of days =	Y□	N□		Central Cyanosis	Y□	N□
Difficulty breathing	Y□	N□	**A & B**	Indrawing	□None/mild □Severe	□Sternum
Diarrhoea: No. of days =	Y□	N□		Grunting	Y□	N□
Diarrhoea bloody	Y□	N□		Air entry bilateral	Y□	N□
Vomits everything	Y□	N□		Crackles	Y□	N□
Difficulty feeding	Y□	N□		Cry	□Normal □Hoarse	□Weak
Convulsions: No. =	Y□	N□	**C**	Femoral Pulse	□ Normal	□ Weak
Partial / focal fits?	Y□	N□		Cap Refill	secs	*X = not possible*
Apnoea	Y□	N□		Murmur Y□ N□	***If yes =***	

**General Examination**				C	Pallor / Anaemia	**0**	**+**	**+++**
Skin	☐Bruising ☐Rash ☐Pustules				Skin cold:	☐Hand ☐Elbow ☐Shoulder		
Jaundice	☐None	☐ **+**	☐**+++**	**Disability**	Can suck / breastfeed?	**Y**☐	**N**☐	
Gest/Size	☐Normal ☐Prem ☐SGA/wasted				Stiff neck	**Y**☐	**N**☐	
Abnormalities – tick all relevant & describe<br>☐Skull =<br>☐Limbs / Spine =<br>☐Palate / Face =<br>☐Genitals / anus =<br>☐Dysmorphic =					Bulging fontanelle	**Y**☐	**N**☐	
					Irritable	**Y**☐	**N**☐	
					Reduced movement / floppy	**Y**☐	**N**☐	
				Umbilicus	☐Clean ☐Local pus ☐Pus + red skin			

Summary

Investigations ordered – record results in medical record

Malaria	☐ Blood slide ☐ Rapid Test	***Glucose***	☐ Stick test ☐ Laboratory
Haematology	☐ Hb ☐ HCT ☐ Full haemogram	***Chemistry***	☐ Na + K ☐ Urea ☐ Creat ☐ LFT
Microbiology	☐ Lumbar Punct ☐ Blood Cult	***HIV***	☐ Rapid test ☐ PCR
X-Ray	CXR ☐ AXR ☐ Other =	***Other 1***	

Admission Diagnoses – Select ONE *primary diagnosis (tick box indicating "1") and ANY secondary diagnoses (tick box indicating "2")*

Birth asphyxia	1 ☐	2 ☐	***Meconium aspiration***	1 ☐	2 ☐	***Maternal HIV Status***	☐Pos ☐Neg ☐Unknown	
Premature / LBW	1 ☐	2 ☐	***Twin delivery***	1 ☐	2 ☐	***Other diagnoses (name below and indicate if primary diagnosis or secondary)***		
Newborn RDS	1 ☐	2 ☐	***Jaundice***	1 ☐	2 ☐		1☐	2☐
Neonatal sepsis	1 ☐	2 ☐	***Meningitis***	1 ☐	2 ☐		1☐	2☐

Supportive care – indicate what care you are providing a plan for and sign please

Vitamin K & TEO	☐	ARVs for PMTCT	☐	**Clinician Name & Sig**
Nutrition / Feeds	☐	Oxygen	☐	
iv fluids	☐	Blood transfusion	☐	
Incubator / Keep warm`	☐	Phototherapy	☐	

Results of Investigations	Date / Test / Results	Date / Test / Results	Date / Test / Results
